# Borrowing from Peter to pay Paul: managing threatened predators of endangered and declining prey species

**DOI:** 10.7717/peerj.7916

**Published:** 2019-10-15

**Authors:** Zeke Davidson, Marc Dupuis-Desormeaux, Arjun Dheer, Laura Pratt, Elizabeth Preston, Saibala Gilicho, Mary Mwololo, Geoffrey Chege, Suzanne E. MacDonald, C Patrick Doncaster

**Affiliations:** 1 Marwell Wildlife, Colden Common, Winchester, Hampshire, United Kingdom; 2 Lewa Wildlife Conservancy, Isiolo, Meru, Kenya; 3 School of Biological Sciences, University of Southampton, Southampton, Hampshire, United Kingdom; 4 Department of Psychology, York University, Toronto, Ontario, Canada; 5 Department of Biology, York University, Toronto, Ontario, Canada; 6 Lewa Wildlife Conservancy Canada, Toronto, Ontario, Canada

**Keywords:** Grevy’s zebra, Lion, Hyena, Endangered species, *Panthera leo*, Wildlife management, Predator

## Abstract

Conservation policy and practice can sometimes run counter to their mutual aims of ensuring species survival. In Kenya, where threatened predators such as lion deplete endangered prey such as Grevy’s zebra, conservation practitioners seek to ensure species success through exclusive strategies of protection, population increase and preservation. We found strong selection for the endangered Grevy’s zebra by both lion and hyena on two small fenced conservancies in Kenya. Despite abundant diversity of available prey, Grevy’s zebra were selected disproportionately more than their availability, while other highly available species such as buffalo were avoided. Lions were therefore not alone in presenting a credible threat to Grevy’s zebra survival. Conservation practitioners must consider interlinked characteristics of prey selection, resource availability and quality, the interplay between carnivore guild members and landscape scale population trends performance in wildlife management decisions.

## Introduction

Range reduction and the fragmentation of wildlife populations threatens biodiversity globally, and links directly to accelerated extinction rates (Fahrig, 2003). As human populations and livestock numbers increase, resources are depleted and their availability to wildlife is reduced; species are not able to adapt fast enough (Chivian & Bernstein, 2008; Ogutu et al., 2016). For vulnerable carnivore-prey populations, increasing predation rates are often assumed to be the proximal causes of species declines, being the most visible signals of change, while the underlying drivers such as rangeland functionality and forage availability may go unnoticed (Ng’weno et al., 2017). The challenges associated with conserving wildlife are compounded in small reserves, when threatened carnivores and endangered prey coexist. Paradoxically, species conservation strategies in Kenya seek to protect and enhance the populations of both carnivore and prey independently (KWS, 2010; KWS, 2018), although these aims cannot be mutually exclusive.

The Grevy’s zebra (*Equus grevyi*) has suffered severe range contractions and population declines owing to human activity in the past 20 years, as with most wildlife generally in Kenya (Ogutu et al., 2016; Rubenstein et al., 2016). The persistence of the Grevy’s zebra population in the study area is important to the survival of the species. An estimated 308 individuals were counted in 2018 (Kaaria et al., 2018; Table S1), down from a high of 632 in 1999, out of 2,250 (range 2,175 to 2,343) individuals detected in the 2016 national census. This amounts to approximately 14% of the Kenyan population (Parham et al., 2017). Despite the small population size of Grevy’s zebra on the Lewa Wildlife Conservancy (LWC) and Borana Conservancy (BC), hereafter the Lewa-Borana Landscape (LBL), they are the third most abundant large mammal in the prey size range for large carnivores in this area (Hayward & Kerley, 2005; Hayward, 2006; Kaaria et al., 2018).

Most of the Kenyan Grevy’s zebra population is found in private or community-owned protected areas such as at our study site. Grevy’s zebra are classified as endangered by the IUCN (Brown & Layton, 2001; Franceschini et al., 2008; Rubenstein et al., 2016). Predation has been cited as a major cause for concern by managers of protected areas hosting the remaining populations and is often seen as the central influence on prey population performance in small reserves (Tambling & Du Toit, 2005; Grange et al., 2012; Miller et al., 2013). For example, Ng’weno et al. (2017) found that in Laikipia, Kenya, lions (*Panthera leo*) affected hartebeest (*Alcelaphus buselaphus*) population performance and suggested that this was exacerbated by environmental change. Unfortunately, they were unable to quantify cumulative predation effect for other members of the large carnivore guild which may further exacerbate population declines. On the LBL, endangered Grevy’s zebra are favoured prey by both lions (*Panthera leo*) and spotted hyenas (*Crocuta crocuta*, hereafter referred to as hyenas), based on carcasses, scat and post hoc analyses of feeding sites identified by GPS telemetry data (Mwololo, 2011; Pratt, 2014; Dheer, 2016; Dupuis-Desormeaux et al., 2015). At our study site, Grevy’s zebras are intensively followed and most animal deaths are recorded. As such, all carcasses are examined to determine the predator involved (Dupuis-Desormeaux et al., 2015). Between 2014 and 2016, there were 43 known adult Grevy’s zebra killed, 31 of these attributed to lions. However, the lion(s) that were responsible for the kills might not defend the carcass for very long given the relatively large hyena population at the study site and the fact that kleptoparasitism is well documented (Périquet, Fritz & Revilla, 2015). Furthermore, carcasses of larger adult prey last longer in the field, and our methods might miss calf predation as these are often consumed and/or dispersed quickly. Therefore, relying solely on carcass data inherently underestimates the kills, though it does provide data on kills that may be underrepresented using other methods such as hair analyses. With this in mind, the objective of our study was to better understand the importance of the Grevy’s zebra to both these apex predators and to elucidate the potential impacts of cumulative predation on Grevy’s zebra population persistence.

Kenya’s wildlife law prevents any form of consumptive measures, including lethal measures, for the management of carnivore population size and structure. Thus, lion management interventions such as those available to wildlife owners in South Africa, including commercial sale, sport hunting and lethal control are not possible under Kenyan legislation (Miller et al., 2013) and are limited to translocation or euthanasia of problem animals. Benefits from wildlife conservation are limited to non-consumptive activities largely represented by photographic tourism. Consequently, on small fenced reserves such as the LBL with high carnivore densities, predation is a complicated and potentially uncontrollable threat to the survival of small populations of preferred prey. This pressure is exacerbated when prey species are also endangered, and populations have been declining steadily for several years, as is the case for Grevy’s zebra on the LBL (Kaaria et al., 2018). Carnivore density is often higher in small fenced areas owing to protection from persecution and conflict. Fences can impede dispersal and cause carrying capacity to be reached quickly (Miller et al., 2013). Fence-gaps designed to encourage wildlife movement and dispersal into safe areas (away from communities) have been shown to be effective on the LBL (Dupuis-Desormeaux et al., 2016b). However, fence-gaps into conservancies are bi-directional and provide a way for predators and prey to return to a conservancy in times of insecurity or poor foraging outside its boundaries. When predator numbers swell due to immigration into a small conservancy, this can destabilize predator socio-spatial behaviour and potentially impact the predator–prey dynamic (Davidson et al., 2011). Determining the dietary habits of carnivores is thus essential to allow informed management decisions at the species level (Kamler et al., 2015). Carnivores that are in the same guild coexist through resource partitioning, with dietary separation being an important factor in facilitating niche separation (Vieira & Port, 2007; Kamler et al., 2012). Behavioural and spatial separation may also facilitate coexistence (Kushata et al., 2018) via different activity peaks (Kamler et al., 2012), foraging behaviour (Périquet et al., 2016), selection for different prey size classes (Purchase, 2004), and segregation of generalist and specialist feeding strategies (Périquet, Fritz & Revilla, 2015). Understanding the relative impacts of carnivores on prey species would help to prioritize management interventions to mitigate population declines.

We investigated carnivore diet selection on the LBL, focusing on two large carnivores, hyenas and lions. However, the conservancy does host the full representation of large East African carnivores, including striped hyena (*Hyaena hyaena*), cheetah (*Acinonyx jubatus*), leopard (*Panthera pardus*) and African wild dog (*Lycaon pictus*). Our first objective was to determine prey selection by both lions and hyenas by investigating dietary contribution. Secondly, we assessed dietary overlap and niche breadth between these species. We used this approach to determine which carnivore species may be responsible for proportionally more impact on the endangered Grevy’s zebra population. We also explored the complexities confounding carnivore management in these challenging circumstances, with relevance to Kenyan conservation policy.

## Materials & Methods

### Study site

**Figure 1:**
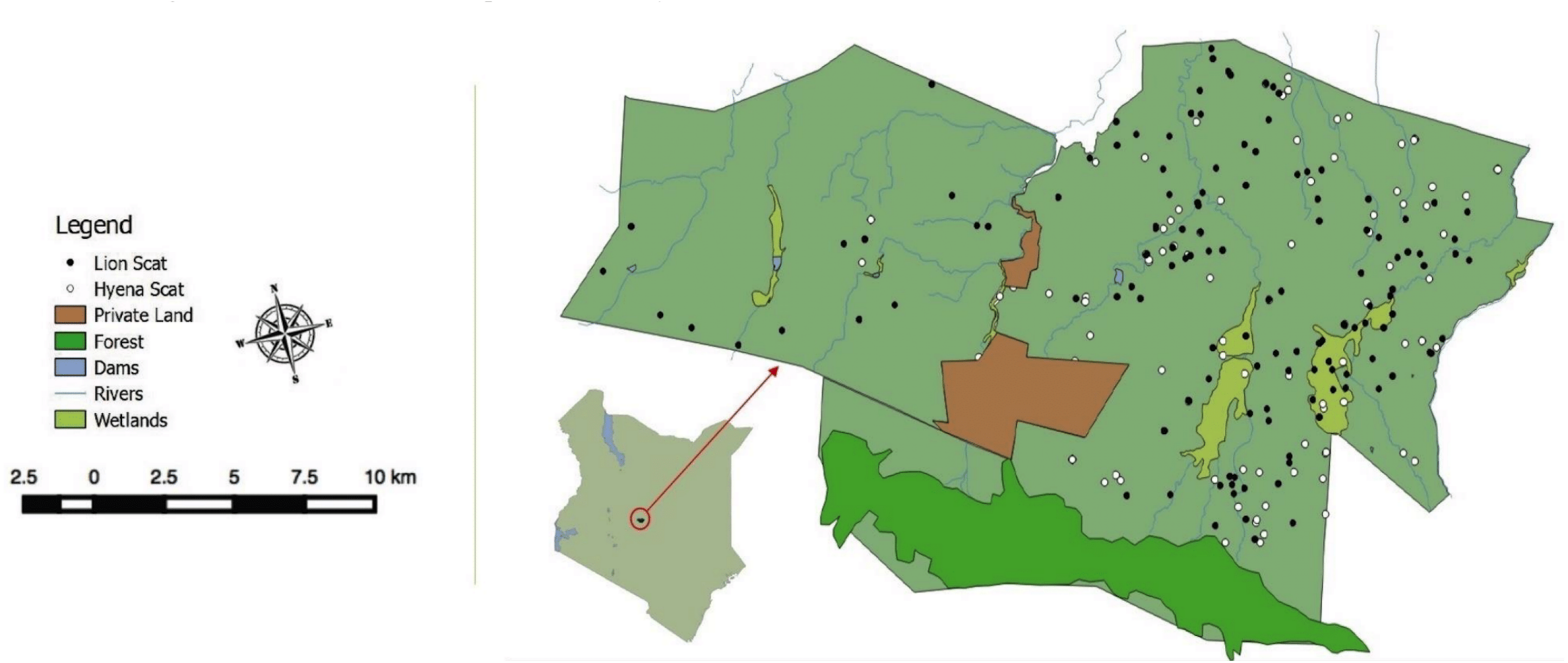
Lewa Borana Landscape (LBL) study area in Kenya, East Africa. Black and white dots represent the locations of recovered lion and hyena scat samples.

We conducted this study at two contiguous properties, Lewa Wildlife Conservancy (LWC) and Borana Conservancy (BC) near Isiolo, Kenya (0.20°N, 37.42°E, the LBL, see Fig. 1). The LBL is a multi-use protected area straddling the border of Laikipia and Meru Counties. The habitat is primarily savannah grassland with interspersed Northern Acacia bushlands and thickets (White, 1983) as well as a network of roads, and fenced agricultural plots, villages, and pasture lands. The combined area of the two conservancies is 375 km^2^. Six migratory gaps are maintained within the combined LBL perimeter fence. The ecosystem allows free movement of all species except for the movements of rhinos, via electric fencing, rocky barriers and wooden post bollards at the migratory gaps (Dupuis-Desormeaux et al., 2016a).

The LBL was originally managed as two livestock ranches. Carnivores were actively encouraged back into the landscape from 1983 and lions were reestablished by 1995 (I Craig, pers. comm., 2016). Some livestock is still maintained within the LBL landscape as part of a holistic vegetation management strategy (Savory & Butterfield, 1998), and to benefit surrounding communities through livestock to market programs. In 2014, the decision was made to remove the fence between LWC and BC in order to create an open landscape that allows for the unrestricted movement of wildlife between the two conservancies (Dupuis-Desormeaux et al., 2018).

The LWC Research Department has monitored Grevy’s zebra and lion populations since 2006, using intensive demographic observations, and scat and kill site-based analysis of lion predation (Mwololo, 2011; Pratt, 2014; Dupuis-Desormeaux et al., 2015). In 2016, hyenas were included in this work through a GPS telemetry study involving five clans that were resident on the LBL (Dheer, 2016).

### Capture and handling of carnivores

The KWS is the authority responsible for all wildlife care and handling in Kenya. All study animals fitted with telemetry devices (collars) were captured, immobilized and handled using standard operating procedures (SOPs) defined and approved by the KWS. Animals were immobilized using a DanInject system, and a mixture of medetomidine and ketamine and monitored closely, visually, until recumbent. While immobilized study animals were monitored physically and physiologically for any adverse effects. Drug reversals were carried out using atipamezole and study animals were closely monitored until they were mobile once again post reversal. This research was carried out under permit number NACOSTI/P/15/8701/5044 National Council for Science, Technology and Innovation, Kenya, and KWS affiliations. All research was conducted at the Lewa Wildlife Conservancy and Borana Conservancy.

### Carnivore density

Lion and hyena densities were known on the LBL based on annual game counts and camera trap data. Sightings records indicated 30 adult lions. In 2018 camera trapping data at active hyena dens on LBL identified 84 adult and 40 subadults and 10 cubs (134 individuals; Table S2).

### Scat analysis

Determination of dietary composition was based on scat analysis (Joslin, 1973). Scat analysis is useful for determining the diets of species that consume readily identifiable plant or animal matter, such as hair (Trites & Joy, 2005; Marucco, Pletscher & Boitiani, 2008; Klare, Kamler & MacDonald, 2011). Given that both hyenas and lions are hypercarnivorous (i.e., >70% of diet is animal matter (Holliday & Steppan, 2004), scat analysis is considered a reliable method to infer their dietary habits through the identification of prey hairs. Both hyena (*n* = 166) and lion (*n* = 144) scats were collected between April 2014 and May 2016. Scats were collected from five hyena clans and four lion prides. Scats were identified by focusing collection at feeding and rest sites identified using GPS cluster analysis (Davidson et al., 2013) and at hyena dens and latrines. Opportunistically encountered scats were also assessed and pooled with the relevant species. Scat of these two species are markedly different and easy to identify visually. Only one fresh scat was collected per site to avoid pseudo-replication. Date of collection was recorded with the GPS location of each sample (Fig. 1; Table S3).

Each scat was placed in a plastic bag for transport to the field laboratory. Scats were air dried in the sun on a mesh surface to permit draining. Dried samples were treated with a mixture of 150 ml 96% ethanol solution and 350 ml boiled water (28.8% solution of ethanol). Twenty hairs were randomly extracted per scat, mounted on microscope slides, and dried overnight. All scat samples were analyzed by the same experienced technician to avoid discrepancies in species identification. Each hair root was compared using medulla cross sections to reference collections compiled in a similar way at the LBL for species identification (Mwololo, 2011; Article S1). Frequencies of different prey hairs were recorded and tabulated for each scat sample.

The occurrence of prey species in scat was used to assess the presence or absence of prey in the carnivores’ diets (Andheria, Karanth & Kumar, 2007). Proportional prey contribution (number of occurrences of species × divided by number of occurrences of all species) was used to standardize the percentages of different prey found in the scat. In the absence of reliably comparable direct observations of carnivores feeding, the frequency of sampled prey hair across scats related to prey abundance was used to determine prey preference (Lyngdoh et al., 2014).

### Prey abundance

Prey species abundance between 2014–2016 was determined based on annual game count data from the LBL. Game counts were conducted systematically by air and vehicle patrol (Kaaria et al., 2018). Livestock counts were not available, so these species (cattle, sheep, and goat) were excluded from abundance-related analyses. However, previous research on the pastoralist communities surrounding LBL revealed that livestock are readily available food sources for carnivores (Oriol-Cotterill et al., 2015) and they were detected in scat samples from both carnivore species in this study.

### Prey selection

Jacobs’ selection index (Jacobs, 1974) was used to assess prey-species selection, in terms of its consumption relative to its availability (Table 1). The index value ranges from −1.0 (avoidance) to +1.0 (selection). The index was calculated for each of the wild prey species for which hairs were detected in scats and for which game count data were available. Chi-square goodness of fit tests were then used to assess whether the observed proportional contribution of the prey species differed significantly from the expected proportional contribution based on the proportion of each prey species in the total prey base. Individual confidence intervals were then calculated for each prey species’ proportional contribution to determine which prey species were causing the possible significance of the chi-square result and whether Jacob’s Index (JI) values with low magnitude were in fact evidence of neutral selection (Neu, Byers & Peek, 1974; Minnie, Boshoff & Kerley, 2015).

**Table 1:** Game count data and proportions of prey hairs in hyena and lion scats. Variables *h* and *l* refer to proportions of prey species in hyena and lion scats, respectively, based on observed data. Bold values indicate cases where the proportion of the prey base that a particular species makes up falls outside the 95% confidence interval.

Prey Species	Census mean 2014–2016	Proportion of prey base	Hyena (*n* = 166)					Lion (*n* = 144)				
			Number of detections	Proportion	Proportion excluding livestock	Jacob’s Index	95% confidence interval	Number of detections	Proportion	Proportion excluding livestock	Jacob’s Index	95% confidence interval
Plains zebra *Equus quagga*	1,268	0.297	154	0.166	0.204	−0.24	**0.193 ≤*h*≤ 0.211**	123	0.215	0.219	−0.20	**0.202 ≤*l*≤ 0.232**
Grevy’s zebra *Equus grevyii*	304	0.071	108	0.116	0.143	0.37	**0.127 ≤*h*≤ 0.158**	95	0.166	0.169	0.45	**0.149 ≤*l*≤ 0.188**
Warthog *Phacochoerus africanus*	83	0.019	11	0.012	0.015	−0.14	0.008 ≤*h* ≤ 0.026	28	0.049	0.050	0.45	**0.035 ≤*l*≤ 0.069**
Buffalo *Syncerus caffer*	978	0.229	83	0.089	0.110	−0.41	**0.093 ≤*h*≤ 0.127**	60	0.105	0.107	−0.42	**0.087 ≤*l*≤ 0.128**
Impala *Aepyceros melampus*	889	0.208	142	0.153	0.188	−0.06	**0.174 ≤*h*≤ 0.198**	40	0.070	0.071	−0.55	**0.054 ≤*l*≤ 0.092**
Kudu *Tragelaphus strepsiceros*	25	0.006	0	0.000	0.000	−1.00	0.000 ≤*h* ≤ 0.006	5	0.009	0.009	0.20	0.003 ≤*l* ≤ 0.021
Giraffe *Giraffa reticulata*	225	0.053	86	0.093	0.114	0.40	**0.097 ≤*h*≤ 0.130**	71	0.124	0.127	0.45	**0.106 ≤*l*≤ 0.147**
Oryx *Oryx besia*	128	0.030	21	0.023	0.028	−0.04	0.018 ≤*h* ≤ 0.041	14	0.025	0.025	−0.09	0.015 ≤*l* ≤ 0.0406
Waterbuck *Kobus elipsiprymnus*	142	0.033	77	0.083	0.102	0.54	**0.086 ≤*h*≤ 0.119**	31	0.054	0.056	0.26	**0.040 ≤*l*≤ 0.074**
Eland *Taurotragus oryx*	231	0.054	73	0.079	0.097	0.31	**0.081 ≤*h*≤ 0.113**	94	0.164	0.168	0.56	**0.147 ≤*l*≤ 0.186**
Cattle *Bos taurus*	NA	NA	68	0.073	NA	NA	NA	11	0.019	NA	NA	NA
Sheep *Ovis aries*	NA	NA	69	0.074	NA	NA	NA	0	0.00	NA	NA	NA
Goats *Capra hircus*	NA	NA	37	0.040	NA	NA	NA	0	0.00	NA	NA	NA
TOTAL	4,273	1	929	1	1	NA	NA	572	1	1	NA	NA

Differences in proportional prey composition between lions and hyena were tested using a Kruskal-Wallis ANOVA test, in which a *p*-value of less than .05 would indicate a significant difference in the predators’ diets.

All statistical analyses were conducted in *R* (R Core Team, 2014).

### Dietary overlap and niche breadth

Pianka’s index (Pianka, 1974) was used to assess dietary overlap between limits of −1.0 (no overlap) and +1.0 (complete overlap). Dietary overlap provides an index of similarity between two carnivores’ diets (Wallace Jr, 1981). Pianka’s index helps to explain mechanisms facilitating coexistence between species in the same guild (Pianka, 1974). It suggests the potential for direct conflict between two carnivores, and to what extent their effects on endangered herbivores can be disentangled from one another by quantifying their proportional reliance on similar prey resources.

Levins’ measure of niche breadth (MacArthur & Levins, 1967; Wallace Jr, 1981) was used to determine the breadth of hyena and lion diets, between limits of 0.0 and 1.0. An index closer to 0 suggests a more diverse diet while one closer to 1 suggests a more specialized diet for a given species. This can have implications for a carnivore’s ability to adapt to fluctuating availability of important prey species (Clavel, Julliard & Devictor, 2011). In general, species with broader diets tend to be more resilient in the face of environmental or anthropogenic disturbance and can readily adopt a variety of feeding strategies (Julliard et al., 2006). Generalist carnivores also may not target specific prey species (e.g., particularly threatened herbivores) as much as their more specialized counterparts (Hayward, 2006), which can mitigate concerns surrounding their feeding behavior.

## Results

Location clustering at carnivore feeding sites did not manifest similarly between lions and spotted hyenas. Lion feeding and resting sites were readily detectable from their location data, allowing lion scats to be easily collected. Feeding sites were not discernable from the hyena GPS movement data. However, clustered hyena locations did allow the detection of latrines and den sites where much of the scat was collected (Fig. 1).

**Figure 2:**
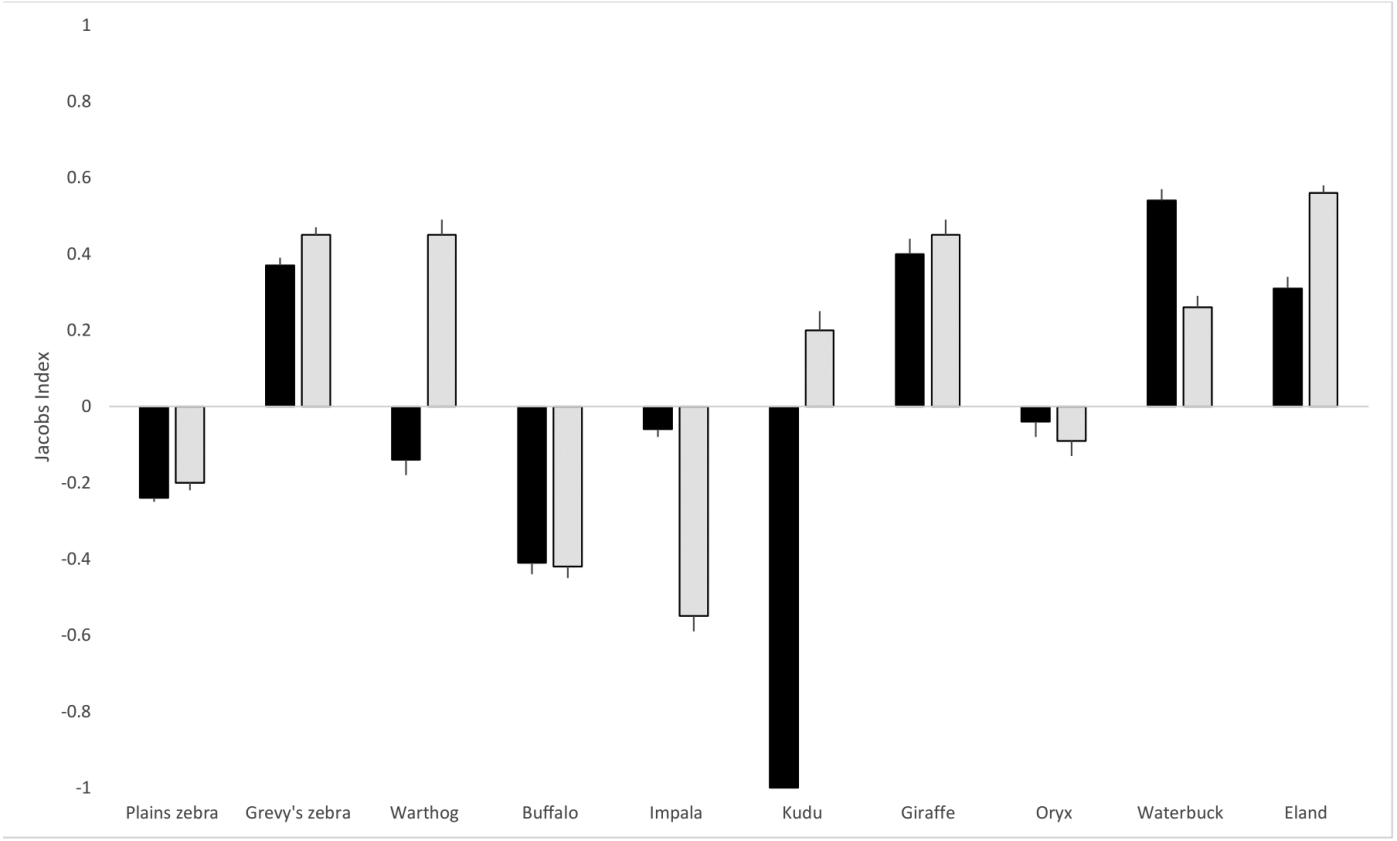
Lion and hyena predation selectivity index. Jacob’s index values (with standard error bars) for lion and hyena prey selection on the LBL. Hyena selection in black bars, lion selection in grey. The index ranges from −1 to +1, where negative values represent relative avoidance and positive values represent relative preference for that prey species.

As shown in Table 1, based on the proportions of hairs detected in scat, the hyena diet was comprised mainly of plains zebra (*Equus quagga*; 16.6%) and impala (*Aepyceros melampus*; 15.3%), and Grevy’s zebra (*Equus grevyi*) contributed a further 11.6%. For lions, plains zebra (21.5%), Grevy’s zebra (16.6%) and eland (*Taurotragus oryx*; 16.4%) were the most frequently detected prey species. Hyenas made use of all three livestock species (cattle, *Bos taurus*; sheep, *Ovis aries*; goat, *Capra hircus*) but lions had a very small proportion (1.9%) of cattle hair in their scat and no sheep or goat hair. Although plains zebra are the most abundant prey species on the LBL, both hyenas and lions exhibited negative selection (JI of −0.24 and −0.21, respectively) for them based on proportional availability and dietary composition (Fig. 2). In the case of Grevy’s zebra, the selection was positive (0.37 for hyena and 0.45 for lion), indicating a distinct selection for this prey compared to its availability. Hyenas also selected for giraffe (*Giraffa reticulata*; 0.40), waterbuck (*Kobus ellipsiprymnus*; 0.54), and eland (0.31), while lions selected for warthog (*Phacochoerus africanus*; 0.45), giraffe (0.45), waterbuck (0.26), and eland (0.56). Of the ten prey species included in the analysis, hyena and lions shared selection in six cases: plains zebra, Grevy’s zebra, buffalo (*Syncerus caffer*), oryx, impala, waterbuck, eland, and giraffe. Variable prey selection was observed in warthog (hyena neutral, lion selecting) and kudu (*Tragelaphus strepsiceros*; hyena avoiding, lion selecting).

For both hyenas and lions, chi-square goodness of fit tests (shown in Table 1) revealed that observed proportions of the prey species differed significantly from expected proportions based on prey abundance (hyena: chi-square = 317.6, *df* = 9, *p* < 0.001; lion: chi-square = 403.1, *df* = 9, *p* < 0.001). Based on the 95% confidence intervals of observed prey detections in the scats, plains zebra, Grevy’s zebra, buffalo, impala, giraffe, waterbuck, and eland were outside the expected confidence range for hyenas. For lions, plains zebra, Grevy’s zebra, warthog, buffalo, impala, giraffe, waterbuck, and eland were outside the expected confidence range. A positive or negative Jacob’s value that is close to 0 may in fact indicate neutral selection, which was the case in hyenas for warthog and oryx and in lions for kudu and oryx.

Pianka’s index of niche overlap for lion and hyena diet was found to be 0.87, indicating the high dietary overlap observed between hyena and lions. Kruskal-Wallis ANOVA further confirmed that hyenas and lions did not differ significantly in proportional prey composition (chi-square = 0.31, *df* = 1, *p* = 0.57). Levin’s index was 0.64 for hyena from 12 consumed species, and 0.48 for lions from ten consumed species, indicating the overall broader diet of hyenas compared to lions on the LBL. Hyenas thus had a wider prey base and more uniform selection across prey species. Species consumed by hyenas that were not consumed by lions included sheep and goats, both domesticated livestock. The only species consumed by lions that hyenas did not consume was kudu, which comprised only a very small proportion of the lions’ diet (0.9%).

## Discussion

Both lions and hyenas significantly prefer Grevy’s zebra, highlighting the potential importance of multi-species predation to the survival of the prey species. This therefore suggests that further investigation to understand the relative impact of each carnivore species on their selected prey is warranted, especially where endangered prey and threatened predators are concerned. In this study we were concerned with the cumulative impact of predation from two top order predators in order to illustrate that predation impact is a complex interaction between several potential predators and their prey. Hence, managing for one predator in an attempt to conserve a prey species is not a sound conservation strategy as predators are not exclusive in their prey selection. Interspecific competition between sympatric predator species is one mechanism that influences prey social structure, spatial distribution, population densities and spatial ecology (Watts & Holekamp, 2008). This has been shown elsewhere in lion/hyena studies (Périquet, Fritz & Revilla, 2015) as well as for other carnivores, for example, between black footed cats (*Felis nigripes*), Cape foxes (*Vulpes chama*), bat-eared foxes (*Otocyon megalotis*), and black-backed jackals (*Canis mesomelas*) in South Africa (Kamler et al., 2015). Although we have direct evidence that lions kill Grevy’s zebra, it is less clear whether hyenas were scavenging or actively hunting Grevy’s zebra. GPS data failed to reveal clustering at hyena kill sites, possibly owing to their rapid feeding behavior which disperses carcass parts quickly and over wide areas (Kolowski et al., 2007). Hyenas are successful predators (Holekamp et al., 1997) and in areas where they greatly outnumber lions, as in the LBL, they may hunt more than lions do (Höner et al., 2002). Further, the Grevy’s zebra is within the potential weight range for hyena prey (Hayward, 2006). Thus, hyenas could potentially be hunting Grevy’s zebra in the LBL. However, even if hyenas are mostly stealing the kills from lions, this kleptoparasitism could trigger more killing of Grevy’s zebra as these are preferred prey of lions. Alternatively, lions might take advantage of hyena hunting success by stealing their kills. As an example of the complex and rapid chain of feeding, we have observed a single lioness killing an adult male Grevy’s zebra only to lose her kill to another lioness within a few hours and in turn that second female losing control of the carcass to a clan of hyenas within the next hour. Regardless, both carnivores feed on Grevy’s zebra regularly and disproportionately more than would be expected in relation to their availability. This may be important in securing the future of zebra populations and more research is required. Specifically, we need research focusing on the entire large carnivore guild instead of past studies that focus on single species, as predation by one large carnivore species may not be significant but cumulative predation threats by multiple species may affect population persistence.

The difference between niche breadth and overlap is important to consider. On the LBL, hyenas have a slightly wider prey niche breadth than lions, meaning they select over a wider range of prey—in this case, by feeding on livestock, possibly when wild prey becomes scarce. In contrast, lions have been shown to focus on selected prey species until decreasing availability precipitates a prey switch (Maruping-Mzileni, Funston & Ferreira, 2017). This typically results in carnivore and prey population sizes cycling (Krebs et al., 2001; Krebs, 2011). Indeed, our data from the LBL indicates that buffaloes are becoming more common in the lions’ diet (LWC research data, unpublished), perhaps due to the increased availability of buffaloes in the landscape owing to population growth and a reduction in Grevy’s zebra availability (Table S1). Meanwhile, the Grevy’s zebra population appears to have settled at a lower level after several years of decline potentially due to factors such as this prey switching. Other carnivores present on the LBL (leopard, cheetah, and wild dog) may also increase the pressure on the Grevy’s population. These demonstrated and possible differences further complicate wildlife management in a system where both predator and prey are vulnerable. Our results differ from O’Brien et al. (2018) where Grevy’s zebra were not selected disproportionately to their availability. However, multiple factors could have contributed to these conflicting results, including the presence of fences and relatively fewer livestock in our study area, and the different methods and focus of the two projects. We further provide a multi-predator, multi-prey analysis which is not the case with either Ng’weno et al. (2017) or O’Brien et al. (2018). We believe both approaches are useful if only to demonstrate that there is no common pathway for managing predation of endangered species, even in closely associated landscapes, in the same county in Kenya. In addition, our analyses do indicate that focusing on one predator instead of the predator guild may miss important information pertaining to cumulative predator effects on threatened prey populations.

Predation threats to managed prey populations can thus produce controversial outcomes, particularly where endangered species are concerned. Furthermore, lions are a threatened species, which further complicates population control. If carnivore control prevents carnivore populations from fluctuating in line with natural prey cycling, management objectives may be undermined by unforeseen consequences in carnivore guild competition and resource selection. In the case of endangered species this might result in the collapse of prey populations on a large scale (Johnson, Isaac & Fisher, 2007). Removing dominant individuals from a population is inherently spatially and socially disruptive (Tuyttens et al., 2000; MacDonald, Riordan & Mathews, 2006; Loveridge et al., 2009; Davidson et al., 2011; Minnie et al., 2018). Furthermore, translocation has yet to be shown as a viable option, due to the territorial nature of pinnacle carnivores and their intense intra- and interspecific competitiveness. Ultimately the effects on the individuals left behind are similar to the impact of lethal measures to achieve the same objective (Fritts, Paul & Mech, 1985). It is understood that translocated individuals do not thrive in new habitats and generally perish post-translocation or return to their native site (Rogers, 1988). Non-lethal interventions to control population size in lions, such as castrating males, is not advised as it produces the same end results as removing individuals: succession, infanticide and population disruption (Miller et al., 2013). There is some evidence that invasive contraception on females can slow population growth to desired levels for small reserve management, without impacting social or spatial relationships (Miller et al., 2013). However, large scale contraceptive methods are not cost effective and require additional intervention to reverse their effects and allow population release at some point.

There does not yet appear to be a safe, viable option for the management of large carnivores. Managing one of the guild members, such as lions, for lower numbers might disturb these relationships, changing their relative abundances and triggering meso-carnivore population release (Trewby et al., 2008). This can potentially cause a sympatric carnivore like hyenas to thrive as has been documented in the Amboseli ecosystem (Watts & Holekamp, 2008). This is true for other carnivore systems such as badgers (*Meles meles*) and red foxes (*Vulpes vulpes*) (Peterson, 1995); and wolves (*Canis lupus*) and coyotes (*Canis latrans*) (Johnson, Isaac & Fisher, 2007). The net result might increase predation threats to selected species and will be unlikely to significantly reduce the overall predation threat to shared prey species like the Grevy’s zebra. In fact, such an intervention may eventually cause general population declines in prey species (Maruping-Mzileni, Funston & Ferreira, 2017).

##  Supplemental Information

10.7717/peerj.7916/supp-1 Table S1 Game count figures on Lewa-Borana Landscape between 2016 and 2018 Collated population sizes for large mammal species on the Lewa-Borana Landscape.

10.7717/peerj.7916/supp-2 Table S2 Population structure of the spotted hyena on the LBL, 2018

10.7717/peerj.7916/supp-3 Table S3 Raw Data for Scat Analysis Raw data comprises a tabulation of all faecal sample analysis for lion and hyena scat collected in the study

10.7717/peerj.7916/supp-4 Supplemental Information 4 The two visible differences between plains zebra hairs and Grevy’s zebra hairs from analysis

## Additional Information and Declarations

### Data Availability

The following information was supplied regarding data availability: Raw data is available in the Supplemental Files. Raw data comprises a tabulation of all faecal sample analysis for lion and hyena scat collected in the study.
