# Supplementary material for: Borrowing from Peter to pay Paul: managing threatened predators of endangered and declining prey species
Source: PeerJ. 2019 Oct 15;7:e7916. doi: 10.7717/peerj.7916 (PMC6798864; doi:10.7717/peerj.7916)
Supplement: Table S1 — Collated population sizes for large mammal species on the Lewa-Borana Landscape. [file peerj-07-7916-s001.docx]

| Species | 2016 | 2017 | 2018 |
| --- | --- | --- | --- |
| Beisa oryx | 179 | 220 | 178 |
| Buffalo | 1220 | 1391 | 1623 |
| Bushbuck | 15 | 17 | 16 |
| Cheetah | 8 | 1 | 4 |
| Eland | 280 | 192 | 322 |
| Elephant | 416 | 509 | 250 |
| Gerenuk | 10 | 10 | 11 |
| Gazelle, Grant’s | 348 | 443 | 415 |
| Gazelle, Thompson’s | 27 | 4 | 7 |
| Giraffe | 273 | 251 | 127 |
| Greater kudu | 28 | 35 | 22 |
| Hippo | 2 | 2 | 2 |
| Hartebeest | 30 | 62 | 64 |
| Hyena, spotted | 0 | 0 | 134 |
| Hyena, striped | 0 | 0 | 12 |
| Impala | 1113 | 1096 | 1763 |
| Jackal (silver backed) | 9 | 12 | 6 |
| Klipspringer | 8 | 8 | 8 |
| Leopard | 7 | 8 | 9 |
| Lion | 17 | 44 | 45 |
| Ostrich | 51 | 44 | 41 |
| Rhino, black | 81 | 82 | 88 |
| Rhino, white | 70 | 75 | 80 |
| Sitatunga | 0 | 0 | 0 |
| Warthog | 68 | 85 | 140 |
| Waterbuck | 136 | 168 | 152 |
| Zebra, Plains | 1262 | 1236 | 1228 |
| Zebra, Grevy’s | 299 | 292 | 308 |

**Table 1: Game count figures on the LBL, 2016-2018.**
