# Supplementary material for: Borrowing from Peter to pay Paul: managing threatened predators of endangered and declining prey species
Source: PeerJ. 2019 Oct 15;7:e7916. doi: 10.7717/peerj.7916 (PMC6798864; doi:10.7717/peerj.7916)
Supplement: Table S2 [file peerj-07-7916-s002.docx]

**Table 2: population structure of the spotted hyena on the LBL, 2018.**

| Clan name | Adults | | | Sub-adults | | | Cubs | | | Total by clan |
| --- | --- | --- | --- | --- | --- | --- | --- | --- | --- | --- |
|  | M | F | Unknown sex | M | F | Unknown sex | M | F | Unknown sex |  |
| Borana | 1 | 0 | 2 | 0 | 0 | 0 | 0 | 0 | 0 | 3 |
| Charlie | 2 | 3 | 16 | 2 | 0 | 16 | 0 | 0 | 0 | 39 |
| Nala | 3 | 3 | 28 | 0 | 0 | 11 | 0 | 0 | 7 | 52 |
| Shamba | 0 | 2 | 8 | 0 | 0 | 4 | 0 | 0 | 0 | 14 |
| Utalii | 2 | 5 | 9 | 0 | 0 | 7 | 0 | 0 | 3 | 26 |
| Total by sex | 8 | 13 | 63 | 2 | 0 | 38 | 0 | 0 | 10 | 134 |
