# Supplementary material for: Borrowing from Peter to pay Paul: managing threatened predators of endangered and declining prey species
Source: PeerJ. 2019 Oct 15;7:e7916. doi: 10.7717/peerj.7916 (PMC6798864; doi:10.7717/peerj.7916)
Supplement: Supplemental Information 4 [file peerj-07-7916-s004.pdf]

The two visible differences between plains zebra hairs and Grevy's zebra hairs from analysis

Scat analysis is typically performed using microscopic photographic analysis of the hair structure. Here below we show a typical photograph of the structure of a hair extracted from carnivore faeces. The lighter outer section is the cortex of the hair and the darker inner section the medulla (Fig. 1).

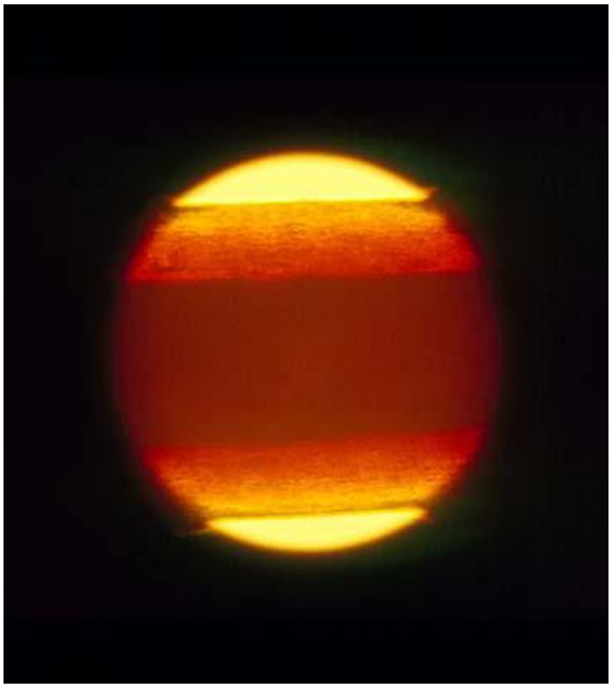

Figure 1: Hair structure from microscopic photography.

Identifying characteristics for comparison between Plains zebra and Grevy's zebra hair

Figures 2 and 3 below show the root section of the two zebra species (plains zebra 2a and Grevy's zebra 2b). Plains zebra hairs typically show a broken medullary core near the root (Fig. 3a) which becomes continuous as it develops toward the tip of the hair, while Grevy's zebra hairs have a solid medulla from root to tip (Fig. 3b).

We developed a methodological operations procedure containing detailed descriptions and clear photographs of each.

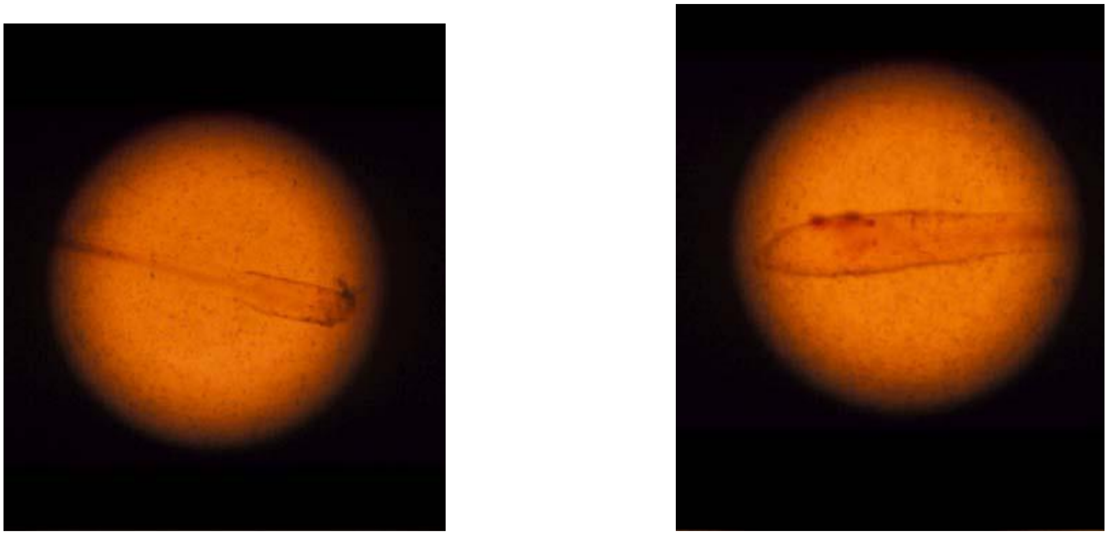

Figure 2: a) Root of plains zebra (PZ) b) Root of Grevy’s zebra (GZ)

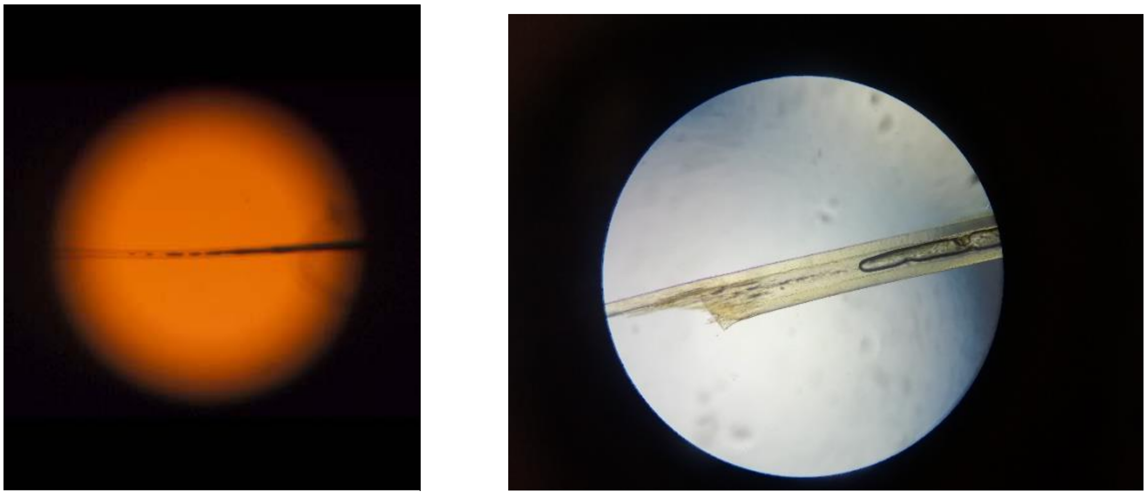

Figure 3: a) Start of medulla of PZ b) Start of medulla of GZ

Examining the medullae of hairs under microscope is a proven and accurate means of species identification (Chernova, 2000). Indeed, relying solely on hair cuticles can lead to misidentification due to damage to the hair while passing through a predator’s digestive tract. Using medullae in addition does not pose this issue because the medullae are located inside the hair cortex and remain protected during digestion (Dharaiya & Soni, 2012). As in Ogara et al. (2010) and De Marinis & Asprea (2006), a reference collection was developed prior to this study with photographs of the hair roots of each prey species in order to establish the unique characteristics pertaining to each prey species’ hair roots (Fig. 2), and medullae (Fig. 3), and the ratio of the hair root width to the cortex. Using this technique allows for economical, efficient, and accurate identification.

References

Chernova OF. Architectonics of the medulla of guard hair and its importance for identification of taxa. InDoklady Biological Sciences 2001 Jan 1 (Vol. 376, No. 1, pp. 81-85). MAIK Nauka/Interperiodica.

De Marinis AM, Asprea A. Hair identification key of wild and domestic ungulates from southern Europe. Wildlife Biology. 2006 Sep;12(3):305-21.

Dharaiya N, Soni VC. Identification of hairs of some mammalian prey of large cats in Gir Protected Area, India. Journal of Threatened Taxa. 2012 Aug 26:2928-32.

Ogara WO, Gitahi NJ, Andanje SA, Oguge N, Nduati DW, Mainga AO. Determination of carnivores prey base by scat analysis in Samburu community group ranches in Kenya. African Journal of Environmental Science and Technology. 2010;4(8):540-6.
